# Supplementary material for: Patterns of Intron Gain and Loss in Fungi
Source: PLoS Biol. 2004 Nov 30;2(12):e422. doi: 10.1371/journal.pbio.0020422 (PMC532390; doi:10.1371/journal.pbio.0020422)
Supplement: Table S1 — Also available at http://genes.mit.edu/NielsenEtAl/. (4.3 MB ZIP). [file pbio.0020422.st001.zip › NielsenEtAl/html/1185.html]

AN5823.1.NCU07117.1.MG04212.1.FG05371.1


```
 CLUSTAL W (1.82) Multiple Sequence Alignments - Introns Inserted


Sequence 1: MG04212.1	568 aa
Sequence 2: FG05371.1	536 aa
Sequence 3: NCU07117.1	520 aa
Sequence 4: AN5823.1	498 aa
Alignment Length: 584 aa
Number Identitical Residues: 176 aa
Alignment Score (without introns) 9486


MG04212.1 	MAPHAEEVLDSSMSSNTSRAPAMSGFSAFLNGSNGANGVNGHHRASNGSNGTNGTVHNGT
NCU07117.1	--------MDDSSSVSSSGYTSEPVANGVSNGSAAATTTSQYKNFS--------------
FG05371.1 	MSPHSEISFSGSDAAVSN--GTSNGINGHSNGTNGFS--NGHMNGHSNGNDAPTAVRHGP
AN5823.1  	--------------MEPLQRKSELDFQSYRKMPLAQQ-----------------------
          	                .    :    ..  : . .                         

MG04212.1 	HNKSRNVPRSQFVLPTPADELHDLICVGFGPASLAIAVAIHDALES-RTLS-----EAPK
NCU07117.1	--------KSRHLRHTPSDEIHDLVCVGFGPASISVAIATHDAIEA-GQLS-----ESPK
FG05371.1 	RVHKE---RSRHLETAPVDSEFDLICAGFGPASLAVAVAMHDAIAEGRNLRP--DGTAPK
AN5823.1  	--------RTQRLKPTSPEELHDLICVGFGPASLAIAIALHDALDPCLNKCAPTSGWQPK
          	        ::: :  :. :. .**:*.******:::*:* ***:       ..:..  **

MG04212.1 	VMFIEKQESFAWHAGMLLPGAKMQISFIKDMASLRDPRSHFTFLNYLHKNGRLVHFTNLD
NCU07117.1	VLFLEKQADFAWHAGMLLPGAKMQISFLKDLASLRDPRSHFTFLNYLHQNDRLIDFINLS
FG05371.1 	VLFLEKQPKFAWHAGMLLPGAKMQISFIKDMATLRNPRSEFTFLNYLHSQGRLVDFTNLG
AN5823.1  	VAFLERQKQFAWHSGMLVPGSRMQISFIKDLATLRDPRSSFTFLNYLHQKDRLIHFTNLS
          	* *:*:* .****:***:**::*****:**:*:**:*** ********.:.**:.* **.

MG04212.1 	TFLPARVEYEDYLRWCASHFEDVVKYSNEAVSVRPEPTPAGGDSSVKIFEVVSRNTKTGV
NCU07117.1	TFTPARVEFEDYLRWCARHFDDVVRYQTEVVAVEPVQSSGP----NKLFTVTSRDVITGE
FG05371.1 	TFLPARTEYEDYLRWCSSWFDHVVNYNNEVLSISPENKEAG---AVKTFTVQARNGKTGQ
AN5823.1  	TFLPARMEFEDYMRWCANQFSDVVTYGEEVIEVLPGKSSPD-SPVVDYFTVLSRNVETGE
          	** *** *:***:***:  *..** *  *.: : *  .    ..  . * * :*:  ** 

MG04212.1 	ETRYRAKNVILALGGQASIPRTLSQAHPKVIHSSQYAHAVPRILRDTSAPYKVAVIGAGQ
NCU07117.1	ITTFQARNVLLALGGQASIPKPLPQKHPRVIHSSQYASVIPKLLRDQSAPVRVAVLGAGQ
FG05371.1 	IQSFRSRHVLVAAGGQPSLPKSLPAKHPRVLHSSQFANYAPQILTKQNAPYRVAVIGAGQ
AN5823.1  	ISSRSARKVVLALGGTAKLPAELPQ-DPRIMHSSKYCTALPNLLKDNNEPYNIAVLGSGQ
          	     :::*::* ** ..:*  *.  .*:::***::.   *.:* . . * .:**:*:**

MG04212.1 	SAAEIFSNVQTLYPNSRTWLVMKSEFLKPSDDSPF2VNSIFNPEFVDNLYPRASSDRRHL
NCU07117.1	SAAEIFNNVQNLYPNSKTYMIMRSEFMKPSDDSPF2VNSIFNPEFIDTIHARPSAYRTHF
FG05371.1 	SAAEIFNNVQNLYPNSKTYLIMRQEFLRPSDDSPF2VNSIFNPEYIDNLWPRSVKARETL
AN5823.1  	SAAEIFHDLQKRYPNSRTSLIMRDTAMRPSDDSPF2VNEVFNPERTDKFYNLSAAERERS
          	****** ::*. ****:* ::*:.  ::******* **.:****  *.:   .   *   

MG04212.1 	IREAKATNYGVVRLELIEKLYELMYDQRRELGSDEKQWPHRIMNSRRVISCEPIKGDRLR
NCU07117.1	LADAKATNYGVVRLELIEHLYEVMYHQRRVLGTDETKWPHRIMAARKVVSVS-EKGDTLK
FG05371.1 	LTEARATNYGVVRLELIEHLFEKMYDQKREISDDETQWPHRILSGREIASVD-TKGDALE
AN5823.1  	LKADKATNYSVVRLELIEEIYHDMYLQR-VKNPDETQWQHRILPSRKITRVE-HYGPNKR
          	:   :****.********.::. ** *:   . **.:* ***: .*.:   .   *   .

MG04212.1 	LRVKPASATADEVEDLQAD-NGEEEEFDADLVVCATGYKRDAHVGMLQSMWGMLPQLNGA
NCU07117.1	IKVGRYFYGEEEETDVTDGPIVDEETLDVDVIICATGYKRMAHVDMLKPTWPLLPEADQA
FG05371.1 	IKVQRVN------DGPLDG-FVDQETFDVDLIIAATGYKRSAHIDMLKDAWTMLPKTVSG
AN5823.1  	MRVHVRAVKDGKDSLIGDG----KEVLEVDALMVATGYNRNAHEQLLSKVQYLRPATQDR
          	::*        .      .    :* ::.* :: ****:* **  :*.    : *     

MG04212.1 	QSTPSEDRKYKVPVFDSWEVQPPADSEDQGTRSLSVGRDYKVRFAPDAVAPGSGVYLQGC
NCU07117.1	LVAEAAKG----SSKDRWFVQGGEE---KSTRVIEANRDYSVKFGEGKVAQGSGIWLQGC
FG05371.1 	RNESPKG-------ANGWNVETQD-----GERKLAVARDYRVKFSPGTVADDSGVWLQGC
AN5823.1  	WTP---------------------------------SRDYRVDLDRSKVSAGAGIWLQGS
          	                                     *** * :  . *: .:*::***.

MG04212.1 	CEGTHG0HYRGLIGEICTY0LGVEVRGAGSPRGLTSER---------
NCU07117.1	CEGTHG0LSDTLLSVLSTR~SGEIVKSIFGA----------------
FG05371.1 	CEGTHG0LSDTLLSVLSTR~SGEIVQSIFKQ----------------
AN5823.1  	NEQTHG~LSDSLLSVLATR~GGEMVESIFGEQLESAAVPDTRFRAML
          	 * ***     *:. :.*   *  *..       ::  ..:   :
```
